# Evaluating the Efficacy of Eravacycline and Omadacycline against Extensively Drug-Resistant *Acinetobacter baumannii* Patient Isolates

**DOI:** 10.3390/antibiotics11101298

**Published:** 2022-09-23

**Authors:** Manas S. Deolankar, Rachel A. Carr, Rebecca Fliorent, Sean Roh, Henry Fraimow, Valerie J. Carabetta

**Affiliations:** 1 Department of Biomedical Sciences, Cooper Medical School of Rowan University, Camden, NJ 08103, USA; 2 Rowan School of Osteopathic Medicine, Stratford, NJ 08084, USA; 3 Department of Medicine, Division of Infectious Diseases, Cooper University Hospital, Camden, NJ 08103, USA

**Keywords:** multidrug resistant, extensively drug resistant, antibiotic resistance, nosocomial, amikacin, eravacycline, omadacycline, bacteria

## Abstract

For decades, the spread of multidrug-resistant (MDR) *Acinetobacter baumannii* has been rampant in critically ill, hospitalized patients. Traditional antibiotic therapies against this pathogen have been failing, leading to rising concerns over management options for patients. Two new antibiotics, eravacycline and omadacycline, were introduced to the market and have shown promising results in the treatment of Gram-negative infections. Since these drugs are newly available, there is limited in vitro data about their effectiveness against MDR *A. baumannii* or even susceptible strains. Here, we examined the effectiveness of 22 standard-of-care antibiotics, eravacycline, and omadacycline against susceptible and extensively drug-resistant (XDR) *A. baumannii* patient isolates from Cooper University Hospital. Furthermore, we examined selected combinations of eravacycline or omadacycline with other antibiotics against an XDR strain. We demonstrated that this collection of strains is largely resistant to monotherapies of carbapenems, fluoroquinolones, folate pathway antagonists, cephalosporins, and most tetracyclines. While clinical breakpoint data are not available for eravacycline or omadacycline, based on minimum inhibitory concentrations, eravacycline was highly effective against these strains. The aminoglycoside amikacin alone and in combination with eravacycline or omadacycline yielded the most promising results. Our comprehensive characterization offers direction in the treatment of this deadly infection in hospitalized patients.

## 1. Introduction

*Acinetobacter baumannii* is an opportunistic human pathogen, which is an aerobic Gram-negative coccobacillus [1]. The manifestations of illness caused by *A. baumannii* predominantly include pneumonia, especially nosocomial pneumonia, catheter-associated bacteremia, and bacteremia secondary to progressed pneumonia. Other illnesses can include soft-tissue infections, urinary-tract infections, and, less commonly, osteomyelitis, endocarditis, meningitis, and necrotizing fasciitis [2,3]. *A. baumannii* infections and outbreaks are becoming increasingly more common worldwide [4]. The United States National Healthcare Safety Network (NHSN) reports that *Acinetobacter* spp. are the cause of 1.8% of all nosocomial infections [5], a rate which is similar to that observed in European and Latin American countries [6,7]. In fact, *A. baumannii* infections in both Asian and some Latin American countries are currently one of the top three most common causes of nosocomial pneumonia and bacteremia [2,8]. *A. baumannii* infections have been linked to the increasing frequency of intensive care interventions, such as mechanical ventilation, central venous catheterization, urinary catheterization, and antibacterial therapy [9]. The World Health Organization (WHO) classified *A. baumannii* as a serious threat [10] and the Center for Disease Control (CDC) classified carbapenem-resistant *A. baumannii* as an urgent threat [10,11].

*A. baumannii* has the highest rate of drug resistance of any Gram-negative pathogen that causes nosocomial infections [5,12,13]. They quickly evolve drug resistance, and multidrug-resistant (MDR), extensively drug-resistant (XDR), and pan-drug-resistant (PDR) isolates have emerged. XDR strains are defined as strains resistant to all systemic antibiotics, with the exception of those that are understood to be less effective or more toxic compared to first-line agents, whereas PDR strains are resistant to every available antibiotic [14,15]. Common antibiotic resistance mechanisms found in *A. baumannii* include alteration of penicillin-binding proteins (PBPs), expression of aminoglycoside-modifying enzymes and beta-lactamases, overexpression of chromosomal efflux systems, and reduction in the permeability of the outer membrane [16]. Imipenem and meropenem are the best treatment options for *A. baumannii* infections, but carbapenem-resistant strains (CRAB) are becoming more prevalent [17,18]. The polymyxins and tigecycline, a third-generation tetracycline-class antibiotic that overcomes most resistance mechanisms, are frequently used as last-line agents; however, resistance to these drugs has also been on the rise. In fact, some studies reported a 50% resistance rate to tigecycline and a 20% resistance rate to the polymyxins [4,19,20]. Therapeutic failure of many of these antibiotics against MDR and XDR strains is leading to increased patient mortality. It is imperative that effective, new treatment strategies for MDR and XDR *A. baumannii* infections are identified, as current treatment options are severely limited.

The development of new antibiotics and subsequent approval is in general a long and slow process. However, new antibiotics have recently been introduced and approved for use in the treatment of certain bacterial infections. Eravacycline, a new fluorocycline, is a broad-spectrum antibiotic that has shown promise in the treatment of complicated intra-abdominal infections [21]. Additionally, in one study, it was demonstrated to be the most effective antibiotic in vitro, when compared to other standard-of-care drugs, against CRAB [22]. Omadacycline is a broad-spectrum aminomethylcycline antibiotic that was recently approved for the treatment of community-acquired (CA) pneumonia and acute skin and skin structure infections (ASSSI, [23]). Since these drugs are newly available, there are limited in vitro data about their effectiveness against either susceptible or MDR *A. baumannii* [22,24]. Additionally, the possibility of treating MDR infections with combinations of new and old drugs has not been adequately explored. One study on the in vitro efficacy of the combination of colistin and eravacycline was evaluated on CRAB and, promisingly, a synergistic effect was reported [25]. More recently, another study found synergistic effects between eravacycline and ceftazidime or a carbapenem against CRAB [26].

Cooper University Hospital experienced an increase in MDR *A. baumannii* colonization and infections from 2004 to 2005, primarily in the intensive care unit (ICU). The goal of this study was to determine the in vitro susceptibility of eravacycline and omadacycline alone and in combination with 22 antibiotics against our *A. baumannii* strain collection. We first characterized the sensitivity of the patient isolates to 22 commonly available standard-of-care antibiotics and found that our collection contains susceptible and XDR strains. We hypothesized that XDR *A. baumannii* would be more susceptible to eravacycline and omadacycline than other tetracyclines and that in combination with other antibiotics, there will be an increase in bactericidal activity. We determined the efficacy of eravacycline and omadacycline against our collection of strains. The entire collection had minimum inhibitory concentrations (MICs) < 4.0 μg/mL for eravacycline, suggesting that this drug may be highly effective in treating infections. For omadacycline, the drug-resistant strains had higher MICs than susceptible strains, suggesting that there may already be some level of tolerance to this drug. As these drugs are relatively new, there are no data available about clinical breakpoints, so firm conclusions cannot be made. We found potential in vitro synergistic effects with eravacycline in combination with amikacin and additive effects with others. We hope that the novel combinations of antibiotics we identified will help inform physician decisions regarding choosing therapeutic agents or provide them new treatment options for patients with difficult-to-treat MDR, XDR, or PDR *A. baumannii* infections.

## 2. Results

### 2.1. Determination of Antibiotic Susceptibilities of Standard-of-Care Drugs against Clinical Isolates of A. baumannii

Our collection of *A. baumannii* strains were collected from patients at Cooper University Hospital (CUH) during a period of increased MDR *A. baumannii* infections that occurred from 2004 to 2005 and includes several sporadic isolates from 2007 to 2012. To begin, we characterized the susceptibilities of 19 selected strains to a full panel of 22 standard-of-care and last-line antibiotics. These strains were isolated at different points in time and displayed different susceptibilities during routine workup performed by the CUH Clinical Microbiology lab. We performed standard broth microdilution assays to determine the MICs and susceptibility to each drug (Table S1). Overall, our collection was highly drug resistant and contained 73.7% XDR and 26.3% susceptible strains, with none being PDR (Table S2). For XDR determination, we used the modern definitions previously proposed for *Acinetobacter* species [14], with removal of the polymyxins based on the recent change in guidelines by the Clinical and Laboratory Standards Institute (CLSI), suggesting that colistin and polymyxin B have limited clinical efficacy [27]. The collection was largely resistant to the individual beta-lactam drugs, including the 3rd- and 4th-generation cephalosporins, β-lactamase inhibitor combination agents, and carbapenems (Table 1). The most effective drugs in these categories were doripenem and cefepime, with 57.9% and 52.6% susceptible strains, respectively. Our collection contained 36.8% CRAB strains, defined as resistant to all three drugs in this class. The overwhelming majority of strains were resistant to tetracycline and the second-generation doxycycline (79.9%, Table 1). However, the majority of strains (73.7%) were susceptible to the third-generation tetracycline class drug, minocycline. In our collection, the resistance rates to the last-line glycylcycline agent tigecycline was 52.6%. In addition, 68.4% and 63.2% of the strains were resistant to the last-line drugs colistin and polymyxin B, respectively. Note that the CLSI no longer reports a susceptible range for these drugs. Among the CRAB isolates, all were colistin resistant and only one was intermediate to polymyxin B (Table S1). The majority of the collection was also resistant to the aminoglycoside gentamicin, while netilmicin and tobramycin were more effective (42.1% susceptible). The aminoglycoside amikacin was highly effective, with 73.7% of strains susceptible (Table 1). The entire collection was resistant to trimethoprim-sulfamethoxazole and the majority were non-susceptible to the fluoroquinolones. The antimycobacterial drug rifampin was somewhat effective, with 52.7% of strains susceptible. Our results suggest that amikacin, minocycline, and doripenem likely would have been effective treatment options for *A. baumannii* infections during these times.

### 2.2. Determination of the Minimum Inhibitory Concentrations of Eravacycline

Eravacycline is a third-generation tetracycline that was recently introduced to the market; thus, there are limited studies on the efficacy of the drug against *A. baumannii* strains [28,29,30,31]. We determined the MICs of eravacycline against our collection of clinical isolates (Table 2). As we do not have any information on clinical breakpoints against *A. baumannii*, either from the CLSI or European Committee on Antimicrobial Susceptibility Testing (EUCAST), we cannot determine if each strain is susceptible or resistant. However, the low MIC values (<4.0) would suggest that eravacycline is effective against our collection of strains. The ranges we observed with our clinical isolates were in agreement with previous reports [28,30,31]. We tested four tetracycline-class drugs against this collection and 26.3% of strains were non-susceptible (intermediate or resistant) to all four drugs (Table S1). Our data suggest that eravacycline overcomes the tetracycline resistance mechanisms present in these strains and could be highly effective for treating *A. baumannii* infections.

### 2.3. Determination of the Minimum Inhibitory Concentrations of Omadacycline

Omadacycline is another newer, third-generation tetracycline-class antibiotic [32]. There have only been a few previous studies on the efficacy of omadacycline against *A. baumannii* [33,34]. We determined the MIC of omadacycline against each strain in our collection (Table 2). The MIC ranges we observed were consistent with previous reports [33,34]. In general, the drug-resistant isolates had higher average MIC values of 6–24 μg/mL, whereas the susceptible isolates had lower MICs of 0.5–4 μg/mL. Once again, there are no clinical breakpoints available from CLSI or EUCAST, so we cannot determine if the isolates are resistant or susceptible. In general, the strains that were susceptible to at least two tetracycline class drugs, had MICs ≤ 4.0. However, as many of the XDR isolates had higher MICs to omadacycline, it is likely that they are intermediate or resistant to this drug, if the cutoff values are similar to other tetracycline-class drugs. Our data suggests that omadacycline may not be effective against strains that already display extensive tetracycline-class drug resistance. More data and available breakpoints are necessary to confirm these observations.

### 2.4. Determination of the Synergistic or Additive Effects of Eravacycline and Omadacycline Combined with Standard-of-Care Antibiotics

**Figure 1:**
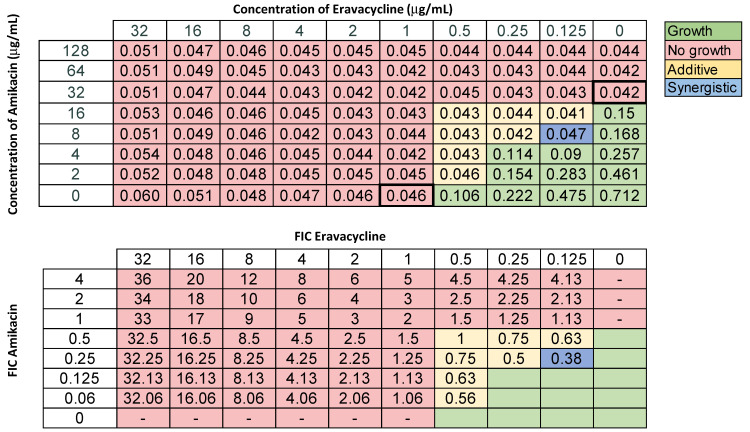
Representative checkerboard assay with eravacycline and amikacin. The XDR strain ACB9 was grown in the presence of varying concentrations of amikacin and eravacycline. Top: OD_600_ measurements following 16 h of static growth at 37 °C. The MICs for each drug alone are outlined with a bold box. The pink boxes indicate wells with no growth (<0.08) and green-colored boxes indicate bacterial growth. The box in the bottom right corner contains no drug and serves as a growth control. Bottom: Fractional inhibitory concentrations (FICs) were calculated for each drug (concentration/MIC) and added together for all wells where no growth was observed. Yellow-shaded boxes indicate additive interactions (FICI between 0.5 and 1.0) and blue boxes indicate synergistic interactions (FICI < 0.5).

In order to study the combinatorial effects of eravacycline and omadacycline, we performed screening disc diffusion assays by placing two antibiotic discs adjacent to each other and visually inspecting them for a larger zone of inhibition between the two discs compared to each drug alone. This indicated a potential synergistic interaction, which was further explored using a checkerboard assay. The MICs were determined and fractional inhibitory concentrations (FICs) calculated, as described in Materials and Methods. For a FIC index (FICI) < 0.5, the combination of antibiotics had a synergistic interaction, while a FICI of 0.5–1.0 indicated an additive effect. For these studies, we selected ACB9, a CRAB isolate that was resistant to nearly every drug screened, including tigecycline and rifampin, but had a low MIC to eravacycline. We tested the combination of eravacycline with amikacin, meropenem, ceftazidime, levofloxacin, ampicillin-sulbactam, and trimethoprim-sulfamethoxazole. We found no combinatorial effects with ceftazidime and additive effects with meropenem, levofloxacin, ampicillin-sulbactam, and trimethoprim-sulfamethoxazole (Figures S1–S4). For eravacycline and amikacin, we found combinations that were additive and synergistic (Figure 1).

**Figure 2:**
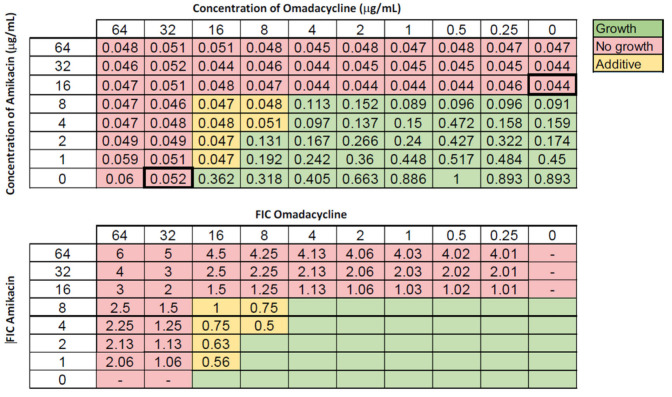
Representative checkerboard assay with omadacycline paired with amikacin. The XDR strain ACB9 was grown in the presence of varying concentrations of amikacin and omadacycline. Top: OD_600_ measurements following 16 h of static growth at 37 °C. The MICs for each drug alone are outlined with a bold box. The pink boxes indicate wells with no growth and green-colored boxes indicate bacterial growth. The box in the bottom right corner contains no drug and serves as a growth control. Bottom: Fractional inhibitory concentrations (FICs) were calculated for each drug (concentration/MIC) and added together for all wells where no growth was observed. Yellow-shaded boxes indicate additive interactions (FICI between 0.5 and 1.0).

All of the most resistant isolates in our collection, including ACB9, displayed increased MICs to omadacycline. However, we identified potential synergistic interactions of various drugs with omadacycline by the disc diffusion screening assays. We tested combinations of omadacycline with ampicillin-sulbactam, tobramycin, ceftriaxone, amikacin, rifampin, piperacillin-tazobactam, and gentamicin. However, following analysis by checkerboard assay, we only found additive interactions with amikacin (Figure 2). All other tested combinations showed no combinatorial effects.

## 3. Discussion

There is an alarming increase in drug resistance among clinical *A. baumannii* isolates. *A. baumannii* strains are now more likely to be resistant to many first-line antibiotics, such as the carbapenems [17], as well as last-line antimicrobial agents [4,19,20]. The rapid emergence of “superbugs” that are MDR, XDR, or PDR [35,36] makes the discovery of new antimicrobial therapies of utmost importance. From 2004 to 2005, Cooper University Hospital experienced an increase in MDR *A. baumannii* infections in the ICU. The strains analyzed contained 36.8% CRAB, 73.7% XDR, and no PDR strains [14]. Interestingly, all 19 of the strains that were tested were resistant to trimethoprim-sulfamethoxazole, suggesting the possibility that these isolates originally evolved from a common ancestral strain. Further evolution likely occurred inside the hospital environment. For the 22 commonly used, standard-of-care antibiotics, the tested isolates were largely resistant to the majority of drugs. This collection of isolates was highly resistant (>70% of strains) to imipenem, ceftazidime, ceftriaxone, doxycycline, tetracycline, gentamycin, and ciprofloxacin. However, the majority of strains were susceptible to the aminoglycoside amikacin and the tetracycline-class drug minocycline, being 73.7% susceptible to each drug (Table 1). Among the CRAB strains, only 42.8% and 28.6% were susceptible to amikacin and minocycline, respectively, with only one strain susceptible to both (Table S1). The next set of drugs that were effective against the majority of strains were doripenem (57.6%), cefepime (52.6%), and rifampin (52.6%). Among the CRAB isolates, 57.1% were susceptible to cefepime and 42.8% were susceptible to rifampin. At CUH, automated antimicrobial susceptibility testing for *A. baumannii* species are performed using the drugs ampicillin-sulbactam, ciprofloxacin, tigecycline, cefepime, gentamicin, tobramycin, trimethoprim-sulfamethoxazole, and meropenem. Amikacin susceptibility is only determined by E-Test when the strains are resistant to tobramycin. If strains are MDR, an E-Test is performed to determine minocycline susceptibility. The E-test, while reliable, requires an additional 16 h of growth. We propose that all strains be tested for amikacin and minocycline susceptibility as part of the primary screening at CUH in order to find effective treatment options for patients and save their lives. We also suggest that the susceptibility to doripenem and rifampin should be determined if treatment options are limited. Our data provides additional treatment options when dealing with MDR or XDR *A. baumannii* infections.

The polymyxins and tigecycline are frequently used as last-line agents, but *A. baumannii* isolates are rapidly emerging that are resistant to these two drugs [19,37,38]. The polymyxins have an adverse patient profile [39], with nephrotoxicity as the most concerning complication, making them an option only in desperate situations. As of 2022, the CLSI no longer reports a susceptible cutoff value for *A. baumannii*, only intermediate and resistant categories for colistin and polymyxin B, due to limited clinical efficacy [27]. Our collection was highly resistant to both of the polymyxin-class drugs (Table 1 and Table S1), with 100% and 85.7% of CRAB isolates resistant to colistin and polymyxin B, respectively. The tigecycline, a broad-spectrum glycylcycline antibiotic, was introduced to the market in 2005 [40]. Tigecycline, like all tetracyclines, targets the ribosome and inhibits translation, but has stronger activity than older-generation drugs and structural modifications to protect against efflux pumps and ribosomal mutations that typically confer resistance [41,42]. However, in our collection, only 31.6% of strains were susceptible to tigecycline and only one was XDR (Tables S1 and S2). Interestingly, the earlier and supposedly less-potent tetracycline-class drug minocycline was highly effective against these isolates. Tigecycline resistance in minocycline-susceptible strains has been observed previously [43]. Perhaps the mechanisms of resistance to these two drugs are slightly different or additive in *A. baumannii* and therefore resistance to each drug should be determined separately and included during routine initial workup. Our data confirms that tigecycline resistance among *A. baumannii* is spreading and is on the rise worldwide [44,45]. 

In 2018, eravacycline, a new tetracycline-class drug, was introduced to the market. This drug was designed to overcome the common tetracycline-resistance mechanisms employed by bacteria [21,28,46]. A 2018 study showed that the in vitro activity of eravacycline was superior against CRAB compared to several other antibiotics [22]. Eravacycline was highly effective against our collection of isolates, with average MICs < 4 for all 19 strains (Table 2), including the XDR ones. This suggests that eravacycline may be a powerful treatment option when faced with MDR or XDR *A. baumannii* infections. As such, it might be worth including eravacycline as part of the routine screening performed in clinical microbiology laboratories during specimen workup. This drug may also be effective against PDR strains, which we could not determine using our collection, but should be assessed. Early studies have indicated that eravacycline is well-tolerated by patients and clinically effective against infections caused by Gram-negative pathogens, including *A. baumannii* [47]. However, eravacycline should be used sparingly in clinical practice and be reserved for highly-resistant bacterial infections. In fact, one potential mechanism of resistance to eravacycline was identified following in vitro evolution experiments and involved overexpression of the AdeABC efflux pump [48]. To overcome any potential resistance development, it might be prudent to use a combination of drugs. We screened novel combinations of drugs and determined that the combination of eravacycline and amikacin resulted in drug synergy (Figure 1). In addition, we found that eravacycline in combination with meropenem, amikacin, levofloxacin, ampicillin-sulbactam, and trimethoprim-sulfamethoxazole had additive effects (Figure 1 and Figures S1–S4). While not a major aminoglycoside-resistance mechanism, mutations in cytochromes or other electron transport chain proteins lower the membrane potential, which reduces aminoglycoside uptake and contributes to overall resistance [49,50,51,52]. If such mutations were present in our strain, this would also reduce the activity of efflux pumps, which are dependent upon the proton motive force (PMF) to function [53]. One possible explanation for the observed synergy is that by decreasing the membrane potential to act against aminoglycosides, the efflux pumps also become impaired, which are major drivers of tetracycline resistance. In other words, altering the PMF to become more resistant against amikacin made the cells more susceptible to eravacycline. We used one XDR strain as a proxy for these experiments and it will be interesting to expand these findings to additional MDR, XDR, or PDR strains to determine if these are general observations. If these results hold true, the combination of eravacycline and amikacin may be a very effective and promising treatment option for patients infected with untreatable isolates of *A. baumannii*.

Omadacycline is a broad-spectrum derivative of minocycline, and is the first aminomethylcycline. This drug has enhanced ribosomal binding and overcomes the common tetracycline-resistance mechanisms [24,54]. In one study, omadacycline was shown to be more active than doxycycline and minocycline against *A. baumannii*, with MICs less than or equal to 4 μg/mL for 90% of the strains [33], the FDA breakpoint for susceptibility of *Klebsiella pneumoniae* to omadacycline. However, among our collection of isolates, we found that only 42% of strains displayed MIC values of ≤ 4.0. This trend suggests that these XDR strains are already intermediate or resistant to omadacycline. A synergistic interaction of omadacycline and amikacin has been previously reported for *A. baumannii*, with 30% of the strains tested showing this pattern [55]. Additionally, synergy has also been reported for omadacycline and meropenem, for 37.5% of the CRAB strains tested [56]. Based on these studies, showing variability among the strains used, expanding this analysis to additional MDR, XDR, or PDR strains is worthwhile. In our study, we did not identify any synergistic combinations, but we did identify several combinations of omadacycline and amikacin that displayed additive effects when using ACB9, which happens to be carbapenem resistant. This finding suggests that further exploration of additional drug combinations with omadacycline are warranted.

Our collection of strains is mostly comprised of XDR isolates; however, our findings showed that eravacycline is highly effective against these strains. As with any drug, it is likely drug resistance will emerge with continued use. To overcome potential resistance development, eravacycline should only be used for virtually untreatable XDR or PDR infections. Second, using combinations of drugs with different mechanisms of action will further protect against resistance development. We identified several additive and synergistic combinations of standard-of-care drugs used with both omadacycline and eravacycline. Some of these combinations may represent powerful new treatment strategies to treat infections with highly resistant strains. Future studies will be aimed at confirming these interactions, exploring new ones, and expanding the analysis to additional strains. If these findings hold true, this suggests that omadacycline and eravacycline may be new weapons in the battle against highly drug-resistant *A. baumannii* infections. Our data arms physicians with new therapeutic options to treat these difficult infections. 

## 4. Materials and Methods

### 4.1. Bacterial Strains, Media, and Growth Conditions

Nineteen *A. baumannii* isolates were collected during routine diagnostic workup at Cooper University Hospital in Camden, NJ, during a period of increased drug-resistant infections from 2004 to 2005, with some sporadic isolates from cases from 2007 to 2012. The isolates were de-identified of all patient information. As the project did not include any interactions or interventions with living individuals or their private identifiable data, the Cooper Human Research Protection Program determined that this project did not meet the definition of human subjects and as a result did not require IRB review. Liquid and agar Mueller–Hinton broth (MHB) were prepared in-house following standard protocols. Strains were inoculated into MHB and grown overnight in a 37 °C shaking incubator. Bacterial growth was monitored by measuring the optical density at 600 nm (OD_600_) when necessary. Antibiotics were purchased as follows: meropenem, imipenem, sulbactam, ceftazidime, tigecycline, doxycycline, colistin, and rifampin from Millipore-Sigma (Burlington, MA, USA); amikacin, minocycline, ampicillin, pipercillin, tazobactam, ceftriaxone, and gentamicin from GoldBio (St. Louis, MO, USA); trimethoprim, tobramycin and tetracycline Bio Basic (Amherst, NY, USA); polymyxin B from Research Products International (Mt. Prospect, IL, USA); levofloxacin and sulfamthoxazole from Tokyo Chemical Industry (Tokyo, Japan); eravacycline and omadacycline from MedChemExpress (Monmouth Junction, NJ, USA); doripenem from Selleck Chemicals (Houston, TX, USA); netilmicin, cefepime and ciprofloxacin from Thermo Scientific (Waltham, MA, USA); cefotaxime from Enzo Life Sciences (Farmingdale, NY, USA); timentin from BioVision (Waltham, MA, USA). Antibiotics were used in amounts and concentrations as indicated below.

### 4.2. Kirby–Bauer Disc Diffusion Assay

Antibiotic disc susceptibility testing was performed in accordance with the American Society for Microbiology’s disc diffusion protocol [57]. Overnight cultures were diluted into 2 mL of fresh MH broth, and allowed to grow at 37 °C for 2–3 h. Following the pre-growth, 300 μL of culture was spread onto a large MH plate. Commercially available discs used were tetracycline, trimethoprim-sulfamethoxazole, gentamicin, levofloxacin, ceftriaxone, and piperacillin-tazobactam (BBL). For all other drugs, the filter paper discs (6 mm diameter) were prepared by adding a specific amount of drug (Table 3), according to the CLSI recommendations [27], and allowing them to dry for at least one hour at 30 °C. The disc diffusion assays were used only as a screen to identify the potential synergistic effects of two drugs, so two antibiotic discs were placed adjacent to each other (about 3 mm apart). Plates were incubated overnight in a 37 °C incubator. A larger zone of inhibition between the two discs compared to each drug alone indicated a potential synergistic interaction, which was then further evaluated using a checkerboard assay.

### 4.3. Broth Microdilution Assay

Determination of the MICs of 22 antibiotics was performed by broth microdilution, according to standard protocols [58]. *A. baumannii* strains were streaked onto MH plates and incubated for 16 h overnight in a 37 °C incubator. A single colony of each strain to be tested was inoculated into 5 mL of MH broth and allowed to grow for 16 h overnight with aeration at 37 °C. The next day, the OD_600_ was determined and cells were diluted into fresh MH broth at a starting OD_600_ of 0.05. Each drug to be tested was added at a 2× concentration, as indicated in Table 4, to the first well of a row in a flat-bottom, 96-well plate. The “X” starting values for each drug was determined based on literature searches or using the data from the CLSI reference standards [27]. Two-fold serial dilutions were performed, and an equal volume of diluted cells added to each well. For polymyxin B and colistin, a final concentration of 0.002% tween-80 was added to each well. The plates were incubated overnight in a 37 °C incubator, without shaking. The following day, the OD_600_ values were read using a Synergy H1 Microplate reader (Biotek). For each strain, the MICs determinations were made at least two independent times.

### 4.4. Checkerboard Assay

The checkerboard assays were carried out as previously described [59,60]. The checkerboard method is similar to that of a standard broth microdilution MIC determination, but the starting concentrations of drugs is 4× (Table 4). It was performed by multiple two-fold serial dilutions of two different drugs in different directions (horizontal and vertical) in a 96-well microtiter plate. Following dilutions, an equal volume of cell culture diluted to an OD_600_ of 0.05 was added and the plates incubated overnight at 37 °C, without shaking. The XDR strain ACB9 was used for all determinations. The OD_600_ was read using the Synergy H1 Microplate reader. The combinatorial effects were estimated by determining various MIC values and calculating the fractional inhibitory concentration index (FICI). The FIC (fractional inhibitory concentration) for each drug was first determined, which is the concentration of the drug divided by the MIC of the drug alone. The FICI was calculated by the following formula: FICI = (MIC A_A+B_/MIC A) + (MIC B_A+B_/MIC B), where MIC A and MIC B denote the MIC of each drug alone and MIC A_A+B_ and MIC B_A+B_ denote the MICs of the drugs in combination. For a FICI < 0.5, the combination of antibiotics has a synergistic effect, while a FICI of 0.5–1.0 indicates additive drug effects. When the FICI is between 1 and 4, the combinatorial effect is indifferent, whereas a FICI > 4 indicates an antagonistic combination. All determinations were made at least two independent times. 

### 4.5. Interpretation of Data

The CLSI breakpoint data were used to determine the antimicrobial susceptibility status of the clinical isolates of *A. baumannii* against most of the studied antibiotics [27]. The proposed susceptibilities of *A. baumannii* for rifampin [61] and tigecycline [62] were used as described. Please note that these values do not represent the true clinical breakpoints as determined by the CLSI. To our knowledge, there is no in vitro breakpoint data for eravacycline or omadacycline against *A. baumannii*.

## Figures and Tables

**Table 1:** Summary of *A. baumannii* susceptibility to standard-of-care antibiotics.

Antibiotic	Susceptible	Intermediate	Resistant
Doripenem	57.9%	5.30%	36.8%
Imipenem	21.1%	0	78.9%
Meropenem	36.8%	0	63.2%
Ampicillin-sulbactam	42.1%	0	57.9%
Piperacillin-tazobactam	31.6%	0	68.4%
Ticarcillin-clavulanate	26.3%	15.8%	57.9%
Cefepime	52.6%	0	47.4%
Cefotaxime	31.6%	0	68.4%
Ceftazidime	21.1%	0	78.9%
Ceftriaxone	26.3%	0	73.7%
Doxycycline	21.1%	0	78.9%
Minocycline	73.7%	10.5%	15.8%
Tetracycline	1.10%	0	98.9%
Tigecycline	31.6%	15.8%	52.6%
Amikacin	73.7%	15.8%	10.5%
Gentamicin	26.3%	0	73.7%
Netilmicin	42.1%	5.30%	52.6%
Tobramycin	42.1%	0	57.9%
Ciprofloxacin	26.3%	0	73.7%
Levofloxacin	26.3%	26.3%	47.4%
Trimethoprim-sulfamethoxazole	0	0	100%
Rifampin	52.6%	0	47.4%
Colistin *	-	31.6%	68.4%
Polymyxin B *	-	36.8%	63.2%

* Based on the 2022 CLSI breakpoint data, there is no susceptible category.

**Table 2:** Average eravacycline and omadacycline MICs (μg/mL).

	Eravacycline	Omadacycline
Strain	MIC	MIC
ACB3	0.06	0.5
ACB4	0.8	6.0
ACB5	0.09	1.0
ACB9	0.69	24
ACB16	3.0	8.0
ACB25	2.1	3.0
ACB28	1.0	16
ACB29	0.8	12
ACB30	2.5	3.0
ACB49	1.0	16
ACB51	3.0	4.0
ACB53	3.0	12
ACB54	0.6	3.0
ACB55	1.2	6.0
ACB56	2.0	4.0
ACB57	0.6	12
ACB58	0.5	2.0
ACB60	0.5	16
ACB61	1.5	8.0

**Table 3:** Quantity of drugs used for disc preparations.

Antibiotic	Amount (μg)
Meropenem	10
Imipenem	10
Ampicillin-sulbactam	10/10
Piperacillin-tazobactam	100/10
Ceftazidime	30
Ceftriaxone	30
Gentamicin	10
Amikacin	30
Tobramycin	40
Tetracycline	30
Doxycycline	5
Minocycline	30
Omadacycline	30
Eravacycline	20
Levofloxacin	5
Trimethoprim-sulfamethoxazole	1.25/23.75
Rifampin	10
Tigecycline	15

**Table 4:** Starting “X” concentration of drugs for the MIC determination.

Antibiotic	X-Value (μg/mL)
Doripenem	32
Meropenem	16
Imipenem	32
Ampicillin-sulbactam	128/64
Piperacillin-tazobactam	512/16
Timentin *	512/34
Cefepime	256
Cefotaxime	512
Ceftazidime	128
Ceftriaxone	128
Gentamicin	128
Amikacin	128
Netilmicin	128
Tobramycin	128
Tetracycline	128
Doxycycline	128
Minocycline	32
Omadacycline	64
Eravacycline	32
Levofloxacin	64
Ciprofloxacin	32
Trimethoprim-sulfamethoxazole	32/608
Rifampin	4
Tigecycline	16
Colistin	64
Polymyxin B	64

* Ticarcillin/clavulanate (15/1).

## Data Availability

Not applicable.

## Supplementary Materials

The following supporting information can be downloaded at: https://www.mdpi.com/article/10.3390/antibiotics11101298/s1, Table S1: MIC determinations for *A. baumannii* strains. Table S2: Resistance classifications of *A. baumannii* strains. Figure S1: Combinatorial effects of eravacycline and ampicillin-sulbactam. Figure S2: Combinatorial effects of eravacycline and levofloxacin. Figure S3: Combinatorial effects of eravacycline and meropenem. Figure S4: Combinatorial effects of eravacycline and trimethoprim-sulfamethoxazole.
