# Supplementary material for: Evaluating the Efficacy of Eravacycline and Omadacycline against Extensively Drug-Resistant Acinetobacter baumannii Patient Isolates
Source: Antibiotics (Basel). 2022 Sep 23;11(10):1298. doi: 10.3390/antibiotics11101298 (PMC9598263; doi:10.3390/antibiotics11101298)
Supplement: Supplementary file 1 [file antibiotics-11-01298-s001.zip › Deolankar et al_Supplement.pdf]

Evaluating the Efficacy of Eravacycline and Omadacycline Against Extensively Drug-resistant *Acinetobacter baumannii* Patient Isolates

Manas S. Deolankar, Rachel A. Carr, Rebecca Fliorent, Sean Roh, Henry Fraimow, and Valerie J. Carabetta

Contents

Figure S1: Combinatorial effects of eravacycline and ampicillin-sulbactam 2

Figure S2: Combinatorial effects of eravacycline and levofloxacin 3

Figure S3: Combinatorial effects of eravacycline and meropenem 4

Figure S4: Combinatorial effects of eravacycline and trimethoprim-sulfamethoxazole 5

Figure S1: Combinatorial effects of eravacycline and ampicillin-sulbactam

Concentration of Eravacycline (μg/mL)

Concentration of Amp-Sul (μg/mL)	32	16	8	4	2	1	0.5	0.25	0.125	0
128/64	0.049	0.056	0.044	0.052	0.047	0.045	0.045	0.045	0.047	0.048
64/32	0.053	0.051	0.043	0.047	0.043	0.043	0.047	0.043	0.08	0.044
32/16	0.049	0.05	0.045	0.045	0.044	0.046	0.047	0.049	0.047	0.053
16/8	0.051	0.046	0.045	0.043	0.043	0.043	0.04	0.096	0.148	0.178
8/4	0.051	0.045	0.046	0.046	0.044	0.044	0.07	0.136	0.205	0.272
4/2	0.051	0.047	0.047	0.041	0.043	0.047	0.05	0.171	0.363	0.572
2/1	0.052	0.049	0.05	0.044	0.043	0.045	0.045	0.458	0.509	0.795
0	0.041	0.048	0.044	0.047	0.043	0.049	0.111	0.684	0.958	0.844

Growth
No growth
Additive
Synergistic

FIC Eravacycline

FIC Amp-Sul	32	16	8	4	2	1	0.5	0.25	0.125	0
4	36	20	12	8	6	5	4.5	4.25	4.13	-
2	34	18	10	6	4	3	2.5	2.25	2.13	-
1	33	17	9	5	3	2	1.5	1.25	1.13	-
0.5	32.5	16.5	8.5	4.5	2.5	1.5	1			
0.125	32.13	16.13	8.13	4.13	2.13	1.13	0.63			
0.06	32.06	16.06	8.06	4.06	2.06	1.06	0.56			
0.03	32.03	16.03	8.03	4.03	2.03	1.03	0.53			
0	-	-	-	-	-	-				

Figure S1. Representative checkerboard assay with eravacycline paired with ampicillin-sulbactam (Amp-Sul). The XDR strain ACB9 was grown in the presence of varying concentrations of amp-sul and eravacycline. Top: OD_{600} measurements following 16 h of static growth at 37°C. The MICs for each drug alone are outlined with a bold box. The pink boxes indicate wells with no growth (<0.08) and green colored boxes indicate bacterial growth. The box in the bottom right corner contains no drug and serves as a growth control. Bottom: Fractional inhibitory concentrations (FICs) were calculated for each drug (concentration/MIC) and added together for all wells where no growth was observed. Yellow shaded boxes indicate additive interactions (FICI between 0.5-1.0).

Figure S2: Combinatorial effects of eravacycline and levofloxacin

Concentration of Eravacycline (μg/mL)

Concentration of Levofloxacin (μg/mL)

	32	16	8	4	2	1	0.5	0.25	0.125	0
64	0.052	0.05	0.053	0.051	0.048	0.04	0.058	0.049	0.047	0.053
32	0.045	0.055	0.047	0.046	0.047	0.046	0.053	0.045	0.044	0.047
16	0.048	0.05	0.048	0.046	0.045	0.045	0.046	0.047	0.044	0.047
8	0.052	0.049	0.046	0.046	0.042	0.044	0.045	0.042	0.043	0.599
4	0.045	0.053	0.045	0.044	0.045	0.05	0.106	0.144	0.264	0.769
2	0.049	0.048	0.045	0.047	0.045	0.051	0.099	0.13	0.358	0.884
1	0.05	0.048	0.047	0.046	0.044	0.052	0.175	0.348	0.371	0.963
0	0.047	0.048	0.048	0.044	0.045	0.051	0.417	0.274	0.493	1.048

Growth
No growth
Additive
Synergistic

FIC Eravacycline

FIC Levofloxacin

	32	16	8	4	2	1	0.5	0.25	0.125	0
4	36	20	12	8	6	5	4.5	4.25	4.13	-
2	34	18	10	6	4	3	2.5	2.25	2.13	-
1	33	17	9	5	3	2	1.5	1.25	1.13	-
0.5	32.5	16.5	8.5	4.5	2.5	1.5	1	0.75	0.625	
0.125	32.13	16.13	8.13	4.13	2.13	1.13				
0.06	32.06	16.06	8.06	4.06	2.06	1.06				
0.03	32.03	16.03	8.03	4.03	2.03	1.03				
0	-	-	-	-	-	-				

Figure S2. Representative checkerboard assay with eravacycline paired with levofloxacin. The XDR strain ACB9 was grown in the presence of varying concentrations of levofloxacin and eravacycline. Top: OD_{600} measurements following 16 h of static growth at 37°C. The MICs for each drug alone are outlined with a bold box. The pink boxes indicate wells with no growth and green colored boxes indicate bacterial growth. The box in the bottom right corner contains no drug and serves as a growth control. Bottom: Fractional inhibitory concentrations (FICs) were calculated for each drug (concentration/MIC) and added together for all wells where no growth was observed. Yellow shaded boxes indicate additive interactions (FICI between 0.5-1.0).

Figure S3: Combinatorial effects of eravacycline and meropenem

Concentration of Eravacycline (μg/mL)

Concentration of Meropenem (μg/mL)	32	16	8	4	2	1	0.5	0.25	0.125	0
16	0.056	0.048	0.046	0.044	0.043	0.046	0.045	0.045	0.044	0.047
8	0.049	0.047	0.045	0.045	0.044	0.043	0.043	0.044	0.044	0.341
4	0.055	0.045	0.045	0.047	0.043	0.051	0.042	0.044	0.047	0.431
2	0.049	0.045	0.045	0.043	0.043	0.044	0.045	0.042	0.181	0.511
1	0.051	0.049	0.045	0.044	0.044	0.044	0.045	0.045	0.179	0.631
0.5	0.051	0.047	0.045	0.043	0.051	0.043	0.043	0.043	0.201	0.558
0.25	0.051	0.046	0.044	0.048	0.045	0.046	0.044	0.043	0.19	0.416
0	0.049	0.049	0.05	0.046	0.044	0.051	0.048	0.042	0.243	0.663

Growth
No growth
Additive
Synergistic

FIC Eravacycline

FIC Meropenem	128	64	32	16	8	4	2	1	0.5	0
1	129	65	33	17	9	5	3	2	1.5	-
0.5	128.5	64.5	32.5	16.5	8.5	4.5	4.5	1.5	1	
0.25	128.25	64.25	32.25	16.25	8.25	4.25	2.25	1.25	0.75	
0.125	128.13	64.13	32.13	16.13	8.13	4.13	2.13	1.13		
0.06	128.06	64.06	32.06	16.06	8.06	4.06	2.06	1.06		
0.03	128.03	64.03	32.03	16.03	8.03	4.03	2.03	1.03		
0.016	128.02	64.02	32.02	16.02	8.02	4.02	2.02	1.02		
0	-	-	-	-	-	-	-	-		

Figure S3. Representative checkerboard assay with eravacycline paired with meropenem. The XDR strain ACB9 was grown in the presence of varying concentrations of meropenem and eravacycline. Top: OD_{600} measurements following 16 h of static growth at 37°C. The MICs for each drug alone are outlined with a bold box. The pink boxes indicate wells with no growth and green colored boxes indicate bacterial growth. The box in the bottom right corner contains no drug and serves as a growth control. Bottom: Fractional inhibitory concentrations (FICs) were calculated for each drug (concentration/MIC) and added together for all wells where no growth was observed. Yellow shaded boxes indicate additive interactions (FICI between 0.5-1.0).

Figure S4: Combinatorial effects of eravacycline and trimethoprim-sulfamethoxazole

Concentration of Eravacycline (μg/mL)

Concentration of TMP-SMX (μg/mL)	32	16	8	4	2	1	0.5	0.25	0.125	0
32/608	0.06	0.068	0.061	0.063	0.064	0.05	0.057	0.07	0.075	0.075
16/304	0.063	0.06	0.048	0.049	0.055	0.052	0.042	0.065	0.064	0.132
8/152	0.055	0.051	0.049	0.047	0.046	0.046	0.045	0.13	0.16	0.143
4/76	0.05	0.047	0.046	0.046	0.048	0.047	0.047	0.165	0.144	0.524
2/38	0.049	0.05	0.049	0.047	0.047	0.046	0.047	0.169	0.265	0.706
1/19	0.07	0.065	0.062	0.06	0.059	0.056	0.055	0.169	0.272	0.759
0.5/9.5	0.07	0.07	0.073	0.073	0.073	0.077	0.057	0.22	0.341	0.775
0	0.07	0.066	0.065	0.063	0.061	0.058	0.054	0.301	0.451	0.922

- Growth
- No growth
- Additive
- Synergistic

FIC Eravacycline

FIC TMP-SMX	64	32	16	8	4	2	1	0.5	0.25	0
1	65	33	17	9	5	3	2	1.5	1.25	-
0.5	64.5	32.5	16.5	8.5	4.5	4.5	1.5	1	0.75	
0.25	64.25	32.25	16.25	8.25	4.25	2.25	1.25			
0.125	64.13	32.13	16.13	8.13	4.13	2.13	1.13			
0.06	64.06	32.06	16.06	8.06	4.06	2.06	1.06			
0.03	64.03	32.03	16.03	8.03	4.03	2.03	1.03			
0.016	64.02	32.02	16.02	8.02	4.02	2.02	1.02			
0	-	-	-	-	-	-	-			

Figure S4. Representative checkerboard assay with eravacycline paired with trimethoprim-sulfamethoxazole (TMP-SMX). The XDR strain ACB9 was grown in the presence of varying concentrations of TMP-SMX and eravacycline. Top: OD_{600} measurements following 16 h of static growth at 37°C. The MICs for each drug alone are outlined with a bold box. The pink boxes indicate wells with no growth and green colored boxes indicate bacterial growth. The box in the bottom right corner contains no drug and serves as a growth control. Bottom: Fractional inhibitory concentrations (FICs) were calculated for each drug (concentration/MIC) and added together for all wells where no growth was observed. Yellow shaded boxes indicate additive interactions (FICI between 0.5-1.0).
